# Preparation and Properties of Intrinsically Black Polyimide Films with CIE Lab Color Parameters Close to Zero and High Thermal Stability for Potential Applications in Flexible Printed Circuit Boards

**DOI:** 10.3390/polym14183881

**Published:** 2022-09-17

**Authors:** Xi Ren, Yan Zhang, Yuang Liu, Changxu Yang, Shengwei Dai, Xiaolei Wang, Jingang Liu

**Affiliations:** School of Materials Science and Technology, China University of Geosciences, Beijing 100083, China

**Keywords:** polyimide, blackness, charge transfer complexes, optical properties, thermal properties

## Abstract

Black polymer films with high thermal stability are highly desired in flexible electrical and electronic fields. Conventional black polymer films based on high-temperature resistant polymers and black inorganic dyes are usually suffered from the poor electrical and tensile properties. In the current work, a series of intrinsically black polyimide (BPI) films with International Commission on Illumination (CIE) Lab optical parameters close to zero and high thermal stability have been designed and prepared. For this purpose, an electron-rich aromatic diamine, 4,4′-iminodianiline (NDA), was copolymerized with 1,4-phenylenediamine (PDA) and 3,3′,4,4′-biphenyltetracarboxylic dianhydride (sBPDA) to afford a series of poly(amic acid) (PAA) solutions, which were then thermally dehydrated to provide the final BPI films at elevated temperatures up to 400 °C in air. The molar fraction of NDA in the total diamine monomers was 0 for BPI-0 (sBPDA-PDA), 10% for BPI-1, 20% for BPI-2, 30% for BPI-3, 40% for BPI-4, 50% for BPI-5, and 100% for BPI-6. For comparison, two referenced polyimide (PI) films, including PI-ref1 and PI-ref2, were prepared according to a similar procedure. The former was derived from pyromellitic dianhydride (PMDA) and 4,4′-oxydianiline (ODA) and the latter was from PMDA and NDA. The BPI films exhibited an increasing degree of blackness with the increasing contents of NDA units in the polymer films. For example, the BPI-6 (sBPDA-NDA) film exhibited the optical transmittance of 1.4% at a wavelength of 650 nm (T_650_), which was obviously lower than those of PI-ref1 (T_650_ = 74.6%) and PI-ref2 (T_650_ = 3.6%). In addition, the BPI-6 film showed the CIE Lab parameters of 0.39 for L*, 2.65 for a*, 0.66 for b*, and haze of 1.83, which was very close to the criterion of “pure blackness” for polymer films (L* = a* = b* = 0). At last, incorporation of the NDA units in the rigid-rod BPI-0 (BPDA-PDA) film slightly deteriorated the high-temperature dimensional stability of the derived BPI films. BPI-6 film showed a linear coefficient of thermal expansion (CTE) value of 34.8 × 10^−6^/K in the temperature range of 50 to 250 °C, which was higher than those of the BPI-0 (CTE = 12.3 × 10^−6^/K), PI-ref1 (CTE = 29.5 × 10^−6^/K), and PI-ref2 (CTE = 18.8 × 10^−6^/K) films. Nevertheless, the BPI films maintained good thermal stability with the 5% weight loss temperatures (T_5%_) higher than 590 °C, and the glass transition temperatures (T_g_) higher than 340 °C.

## 1. Introduction

Polyimide (PI) films have been widely used in modern industry for more than half a century since their first commercialization in 1960s due to the excellent combined thermal, mechanical, dielectric properties, and good environmental stability [1,2,3]. It has been well-established that the strong inter-molecular and intra-molecular charge transfer (CT) interactions from the electron-donating diamine units to the electron-accepting dianhydride units play very important roles for the above-mentioned excellent properties of the standard wholly aromatic PI films [4,5,6]. Thus, in theory, the properties of PI films could be tailored by adjusting the degree of CT interactions in the polymers. One of the most successful cases for functionalizing the PI films by adjusting the CT interactions is the research and development of colorless and transparent PI films in the past decades [7,8,9]. Researchers successfully reduced the absorption of visible light in the PI films by inhibiting or weakening the CT interactions inside the PI films, thus regulating the colors of PI films from traditional brown-yellow to pale-yellow, or even to colorless [10,11,12]. Contrarily, it could be anticipated that if the CT interactions inside the molecular structures of the PI films were enhanced, it might be possible to deepen the colors of the PI films, even to be totally black.

Black PI (BPI) films represent a class of special PI films characterized by the black appearance, which have been widely used in the fabrication of flexible printed circuit board (FPCB) in semiconductor industry, as voice coils in loudspeakers, or as thermal control components in aerospace vehicles [13,14,15]. The black appearance designed for the BPI films for the above-mentioned applications is mainly based on the consideration of shielding (intellectual property rights protection, etc.), covering (ultraviolet-light shielding, etc.), heat controlling (temperature adjusting, etc.), or other purposes. As we know, the standard PI films, such as the commercially available Kapton^®^ (trademark of DuPont, USA) film based on pyromellitic dianhydride (PMDA) and 4,4′-oxydianiline (ODA) or Upilex^®^-S (trademark of Ube Industry, Japan) film derived from 3,3′,4,4′-biphenyltetracarboxylic dianhydride (sBPDA) and 1,4-phenylenediamine (PDA), or Upilex^®^-R (trademark of Ube Industry, Japan) film derived from sBPDA and ODA, usually showed the colors from dark-brownness to deep-yellowness due to the strong CT interactions in the highly conjugated molecular chains [16]. However, the CT interactions are usually not enough to endow the PI films with the black colors. Thus, in order to achieve the desirable blackness for the PI films, various methodologies of blending with fine carbon powders or other black additives have to be adopted in practical applications [17,18,19,20,21,22]. What is more, the loading amounts of the black additives are usually higher than 10 wt% so as to obtain the desired blackness. The BPI composite films usually possess deteriorated electrical and mechanical properties at such high contents of the black additives [23].

**Figure 1:**
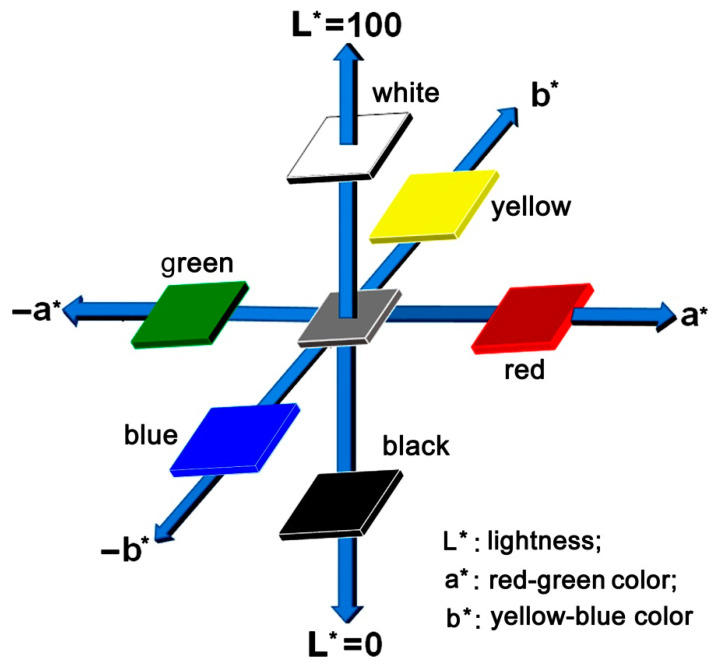
Diagrammatic CIE Lab optical parameters.

In recent years, the BPI films with intrinsically black or deep appearances and the good electrical, thermal, and mechanical properties have attracted increasing attention from the academic and engineering areas. Most of the works have been focused on the incorporation of the third or more diamine components with enhanced electron-donating features, such as the diamines containing quinoxaline [24,25], anthraquinone [26], and other structural units. For example, very recently, Zhou et al. reported the intrinsically black PI films derived from 2,4,5,7-tetraamino-1,8-dihydroxyanthracene-9,10-dione (4NADA) diamine [26]. The copolymerized Kapton-type PI films containing 4NADA with the molar ratio of 4% in the diamine moieties showed the lowest CIE Lab optical parameters of L* = 20.8, a* = 0.8, and b* = −2.6. In our precious study, a series of intrinsically BPI films were developed from a nitrogen-bridged aromatic diamine, 4,4′-iminodianiline (NDA) [27,28]. Low CIE Lab optical parameters were achieved for the derived BPI film based on PMDA and NDA with the curing temperature as high as 350 °C [27]. In our experiments, the cooperative effects of the enhanced CT interactions and the high-temperature micro-oxidization of the imino (–NH–) linkages in the BPIs were found to contribute to the blackness of the afforded BPI films. According to the definition of CIE Lab color parameters shown in Figure 1 (L^*^ is the lightness, where 100 means white and 0 implies black. a^*^: positive value means red, negative value indicates green; b^*^: positive value means yellow, negative value indicates blue), the ideal “pure blackness” for the polymer films should be L* = 0. Meanwhile, in order to eliminate the effects of other colors, the a^*^ and b^*^ values of the polymer films are also preferred to be 0. Thus, the PI films with intrinsically pure blackness usually showed the ideal feature of L* = a* = b* = 0. In practice, this “pure blackness” for pristine polymer films is usually impossible to be achieved due to the intrinsic light-transmitting characteristics for the organic polymers, especially for polymer films with thin thickness.

In the current work, as an effort to explore the limitations of the blackness for polymer films via the structural modification, a series of BPI films were designed and developed based on the highly conjugated poly(biphenyltetracarboxylic dianhydride-1,4-phenylenediamine) (BPI-0) matrix. NDA was incorporated into the molecular structure of BPI-0 via copolymerization. Meanwhile, a high-temperature imidization procedure up to 400 °C in air was used for the preparation of the BPI films. Effects of the NDA units and the high-temperature imidization on the thermal and optical properties of the BPI films were investigated in detail.

## 2. Materials and Methods

### 2.1. Materials

Highly pure (purity ≥ 99.5%) 3,3′,4,4′-biphenyltetracarboxylic dianhydride (sBPDA) was purchased from Guchuang New Chemical Materials Co., Ltd. (Shanghai, China) and dried at 180 °C in vacuo for 24 h prior to use. 1,4-Phenylenediamine (PDA) was purchased from Tokyo Chem. Ind. Co., Ltd. (TCI, Tokyo, Japan) and used as received. The white crystals of 4,4′-iminodianiline (NDA) were prepared in our laboratory and recrystallized from aqueous ethanol in a glove box before use. Ultra-dry N-methyl-2-pyrrolidinone (NMP), with the water content lower than 50 ppm, was purchased from InnoChem Sci. Technol. Co., Ltd. (Beijing, China). The other commercially available reagents were purchased and used as received.

### 2.2. Measurements

A DV-II+ Pro viscometer (Brookfield, Ametek, Middleborough, MA, USA) measured the absolute viscosity of the poly(amic acid) (PAA) solutions at 25 °C. The number average molecular weight (M_n_) and weight average molecular weight (M_w_) of the PAA varnishes were tested with a gel permeation chromatography (GPC) system (Shimadzu, Kyoto, Japan). The Fourier transform infrared (FTIR) spectra of the PI films were measured on an Iraffinity-1S FT-IR spectrometer (Shimadzu, Kyoto, Japan). Ultraviolet-visible (UV-Vis) spectra of the samples were recorded on a U-3210 spectrophotometer (Hitachi, Tokyo, Japan). Wide-angle X-ray diffraction (XRD) was conducted on a D/max-2500 X-ray diffractometer (Rigaku, Tokyo, Japan). CIE Lab color parameters of the BPI films were measured using an X-rite color i7 spectrophotometer (Grand Rapids, MI, USA) with PI films at a thickness of 25 μm. The color parameters were calculated according to a CIE (International Commission on Illumination) Lab equation. L^*^ is the lightness, where 100 means white and 0 implies black, as shown in Figure 1. The whiteness indices (WI) of the BPI films were calculated as follows: WI = 100 − [(100 − L*)^2^ + a*^2^ + b*^2^]^1/2^.

Thermogravimetric analyses (TGA) of the BPI films were measured on a TA-Q50 thermal analysis system (New Castle, DE, USA) at a heating rate of 20 °C/min in nitrogen. Dynamic mechanical analyses (DMA) were tested with a TA-Q800 thermal analysis system (New Castle, DE, USA) with a heating rate of 5 °C/min and a frequency of 1 Hz in nitrogen. Thermo-mechanical analysis (TMA) measurements were carried out on a TMA402F3 thermal analysis system (NETZSCH, Selb, Germany) in nitrogen with a heating rate of 5 °C/min. The coefficients of linear thermal expansion (CTE) values of composite films were recorded in the range of 50–250 °C.

### 2.3. Preparation of PI films

A standard two-step procedure was used for the preparation of the BPI films. BPI-6 was used as an example to illustrate the preparation pathway. For the poly(amic acid) (PAA) precursor synthesis, NDA (19.9250 g, 100 mmol) and DMAc (148.0 g) were added into a 500 mL flask equipped with a mechanical stirrer, a cold water bath, and a nitrogen circulating system. The diamine solution was cooled to be 5–10 °C. Although the diamine solution was carefully protected with nitrogen to prohibit the oxidation, the color of the solution turned from colorless to purple within 10 min. Then, sBPDA (theory amount: 29.4220 g, 100 mmol) was gradually added to the NDA solution and the absolute viscosity of the PAA solution increased gradually. When the absolute viscosity of the PAA solution reached 10000 mPa s, the addition of sBPDA ceased. The amount of BPDA was found to be 29.1337 g, which was about 99.0% of the theoretical amount. Then, the PAA solution was stirred at 5–10 °C for 24 h under nitrogen. The obtained black and viscous solution with the solid content around 25 wt% was diluted with DMAc to afford the homogeneous PAA solution with the solid content of 15 wt% and viscosity around 6000 mPa s.

The PAA solution was then filtered through an automatic filter press with the filter membranes of 1.0 μm to remove any impurities. Then, the purified PAA solution was cast onto a clean glass substrate and thermally baked in a high-temperature oven in air conditions according to the following imidization procedure: 80 °C/2 h, 150 °C/1 h, 250 °C/1 h, 350 °C/1 h, and 400 °C/1 h. Then, the temperature was decreased naturally to room temperature. The glass substrate was immersed into deionized water preheated to 80 °C. Then, free-standing BPI-6 film peeled off the substrate and was obtained as a flexible and tough film with a black appearance.

The other BPI films were prepared according to similar procedures, as mentioned above. In addition, two referenced PI films were also prepared from pyromellitic anhydride (PMDA) with 4,4′-oxydianiline (ODA) for PI-ref1 [27] and with NDA for PI-ref2 [27] according to the procedure reported in our previous work. The properties of the PI-ref films were correspondingly cited in the current work.

## 3. Results and Discussion

### 3.1. PI Films Preparation

**Figure 2:**
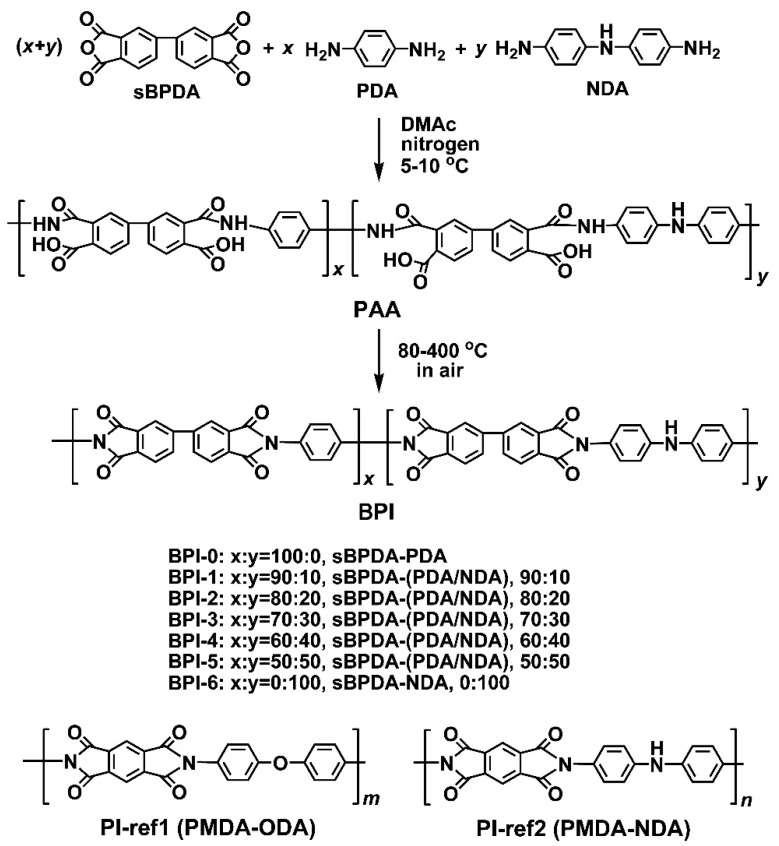
Preparation of BPI (BPI-0~BPI-6) and PI-ref (PI-ref1 and PI-ref2) films (x, y, m, n represent the numbers of repeating units for the polymers).

**Figure 3:**
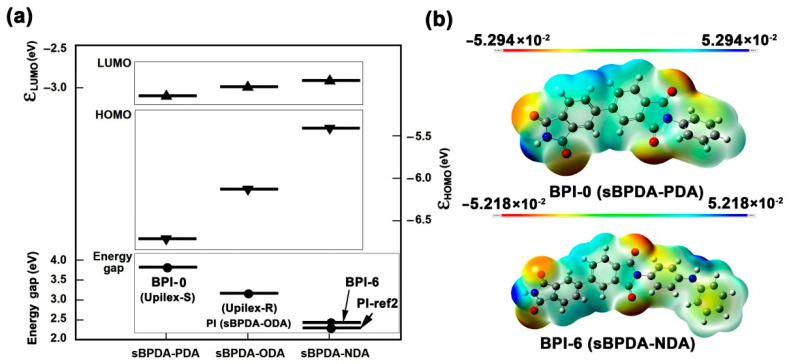
Calculations for PIs via B3LYP/6-311G(d,p) model. (**a**) Molecular orbital energy; (**b**) electrostatic potential map.

The BPI films were designed and prepared via a well-established two-stage polymerization procedure for PI films preparation shown in Figure 2. The soluble PAA precursors were first synthesized and then thermally imidized to afford the final PI films at elevated temperature up to 400 °C in an air environment. BPI-0 and BPI-6 were homopolymers and represented the two ends for the series of polymers, while BPI-1–BPI-5 were copolymers with tailored molar ratio of PDA and NDA diamines. It has been mentioned in the Introduction section that the colors of the PI films are highly affected by both of the CT interactions in the polymer chains and the processing conditions for the films. The CT interactions could be roughly estimated by the energy level gap (E_g_) between the lowest unoccupied molecular orbital (LUMO) energy levels (ε_LUMO_) and the highest occupied molecular orbital (HOMO) energy levels (ε_HOMO_) for the repeating units of the PIs (E_g_ = ε_LUMO_−ε_HOMO_) [29,30,31]. The ε_LUMO_ and ε_HOMO_ values of the PI molecular chains could be calculated according to the density functional theory (DFT)/B3LYP methods with Gaussian 09 software using the 6-311G(d) basis set [29]. Generally, the lower the E_g_ values, the more significant the CT interactions in the polymers, and the more significant the absorption of visible light by the polymers. The calculated molecular orbital energy and electrostatic potential maps of PIs are shown in Figure 3. The PIs showed a decreasing E_g_ value of BPI-0 (sBPDA-PDA) > PI (sBPDA-ODA) > BPI-6 (sBPDA-NDA), indicating that BPI-6 possessed much stronger CT interactions in the molecular chains than that of BPI-0. This is mainly due to the enhanced CT interactions by the lone pair of electrons in the nitrogen in NDA units.

The molecular weights of the PAA precursors are shown in Table 1. In the current research, the final absolute viscosities were controlled to be around 6000 mPa s in order to meet the future fabrication procedure for FPCBs. Thus, the molecular weights of the PAA precursors were adjusted by varying the molar amounts of sBPDA. The PAA varnishes exhibited the number average molecular weights (M_n_) and polydispersity indices of 2.73 × 10^4^–3.34 × 10^4^ g/mol and 1.53–3.18, respectively. Although the molecular weights of the PAA vanishes were controlled in order to achieve a relatively low viscosity at a high solid content, the moderate M_n_ values were expected to meet the requirements of the following PI film preparations. In addition, it could be noticed that incorporation of the NDA components increased the PDI values of the PAAs, which might be due to the somewhat branching reactions between the highly reactive anhydride groups in sBPDA and the low-reactive imido (-NH-) groups in NDA units during the polymerization.

**Figure 4:**
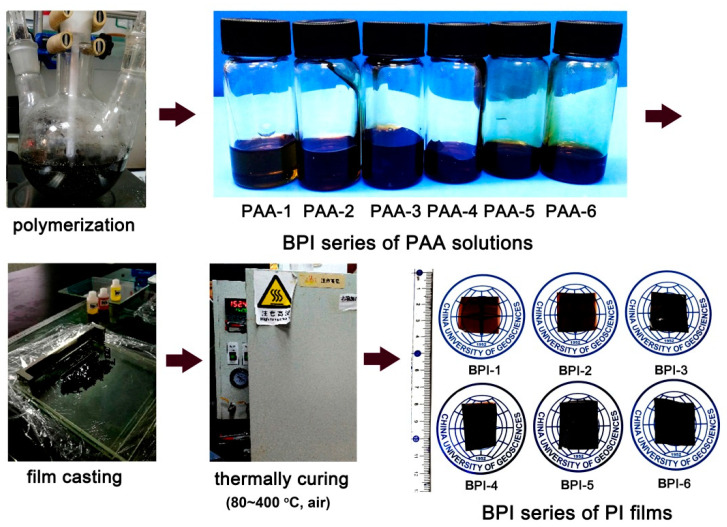
Preparation procedure for the BPI films.

**Figure 5:**
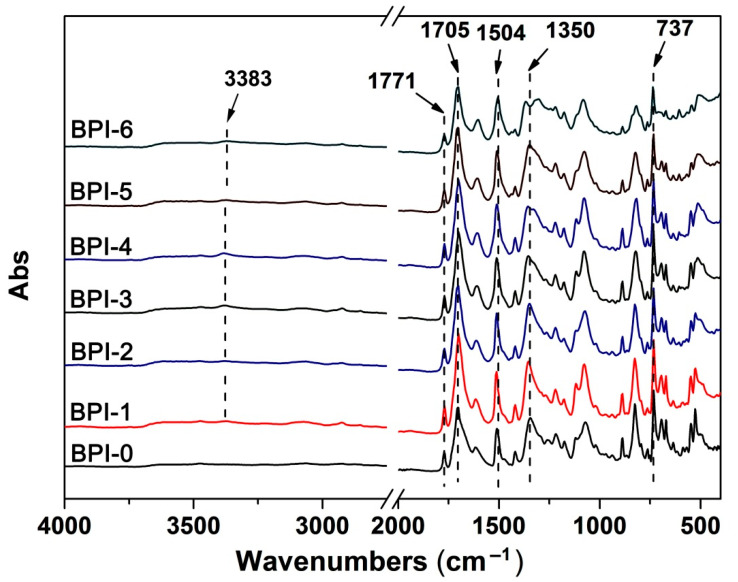
FTIR spectra of BPI films.

As illustrated in the procedures shown in Figure 4, a series of BPI films were prepared by thermally imidized the PAA precursors in the temperature range of 80–400 °C. It could be clearly observed that the colors of the PI films gradually blackened with the increasing of the NDA contents in the polymers. When the molar ratio of NDA in the total diamines reached 40% (BPI-4), the films became totally black in the color. FTIR measurements were performed to confirm the chemical structures of the BPI films and the results are shown in Figure 5. First, the imide rings revealed a series of characteristic absorptions in the spectra for all of the polymers. For instance, the imide carbonyl groups revealed the asymmetric and symmetric stretching vibrations at 1771 cm^−1^ and 1705 cm^−1^, respectively. They also showed the bending vibrations at 737 cm^−1^. The imide C-N groups showed the stretching vibrations at 1350 cm^−1^. In addition, the C=C bonds in phenyl ring exhibited the characteristic absorptions at 1504 cm^−1^. The weak characteristic absorptions of N-H stretching vibrations at 3383 cm^−1^ were only observed for BPI-1–BPI-6. The FTIR information revealed here confirmed the successful preparation of the films.

### 3.2. Optical Properties

**Figure 6:**
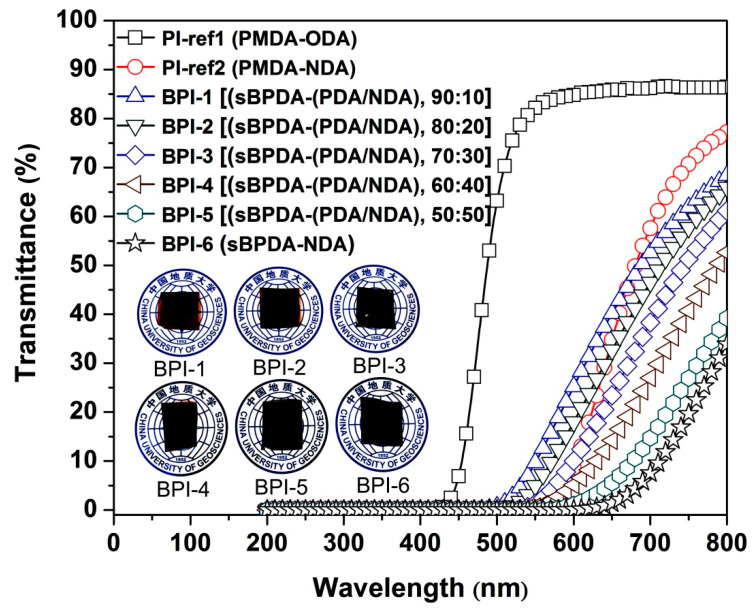
UV-Vis plots of the BPI and PI-ref films (Insert: appearance of the BPI films).

One of the main targets for the current work was to develop PI films with intrinsically black colors close to “pure blackness”. Two pathways were adopted for this target, including the enhancement of CT interactions in the PI (sBPDA-PDA) system by the lone pair of electrons containing NDA diamine and the slight oxidation of the –NH– groups at high temperatures. UV-Vis and CIE Lab measurements were performed to evaluate the structure–optical properties relationship of the afforded BPI films. The results are listed in Table 2. The UV-Vis spectra (Figure 6) of the PI films indicated that the developed BPI films exhibited quite low optical transmittance in the visible light regions. They showed the cutoff wavelengths (λ_cut_) in the range of 415–572 nm, which were lower than that of the standard PI-ref1 (PMDA-ODA) film (λ_cut_ = 407 nm). In addition, the λ_cut_ values of the BPI films increased with the increase of the NDA contents in the films. The optical transmittances of the BPI films at different wavelengths of 550 nm (T_550_), 650 nm (T_650_), and 760 nm (T_760_) were also recorded. The BPI-6 film showed the T_550_, T_650_, and T_760_ values of 0, 1.4%, and 22.5%, respectively, which were obviously lower than those of the PI-ref1 film (T_550_ = 70.4%; T_650_ = 74.6%; T_760_ = 80.9%) and were comparable to those of the PI-ref2 (PMDA-NDA) films cured at 400 °C (T_550_ = 0; T_650_ = 3.6%; T_760_ = 36.8%). The effects of the curing temperature on the optical properties of the PI films could be revealed by comparing the optical transmittances of the films at different curing temperatures. For example, it could be seen from the data shown in the parentheses that the 350 °C-cured PI-ref2 film showed the T_550_, T_650_, and T_760_ values of 0.4%, 34.8%, and 72.4%, respectively, which were apparently higher than those of the 400 °C-cured counterparts. It should be noticed that the curing condition of 400 °C in a natural environment is quite common in the practical manufacturing of commercial PI films, especially for the biphenyl types of PI films [32,33]. Thus, the currently used film-making conditions at a high temperature of 400 °C are reasonable and acceptable for the industrial applications.

**Figure 7:**
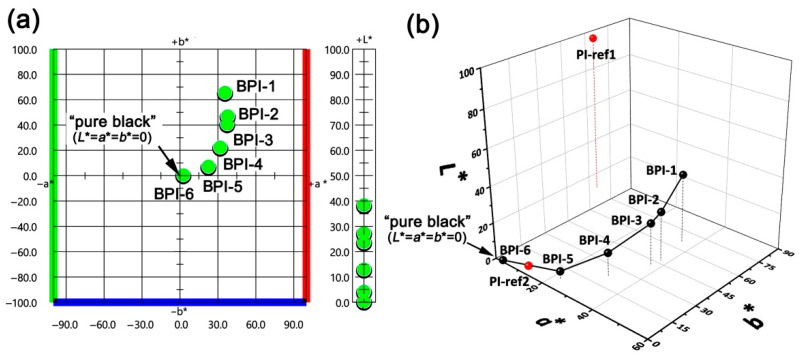
CIE Lab color parameters of the BPI and PI-ref films. (**a**) 2D image; (**b**) 3D image.

The CIE Lab color parameters of the BPI films were measured, and the plots are shown in Figure 7. All the BPI films exhibited the lightness (L*) values below 40, indicating the dark nature of the films. BPI-6 film had a L* value of 0.39, which was much lower than that of the PI-ref1 (L* = 88.65) and very close to the pure blackness (L* = 0). Meanwhile, the BPI-6 film showed the positive a* and b* values of 2.65 and 0.66, respectively, indicating a little bit red of and yellow nature in the film. The whiteness index (WI) of the BPI-6 film was only 0.35. In addition, the BPI-6 film showed the low haze of 1.83%. Thus, BPI-6 could be a film with the degree of blackness close to “pure black” level (L* = a* = b* = 0).

**Figure 8:**
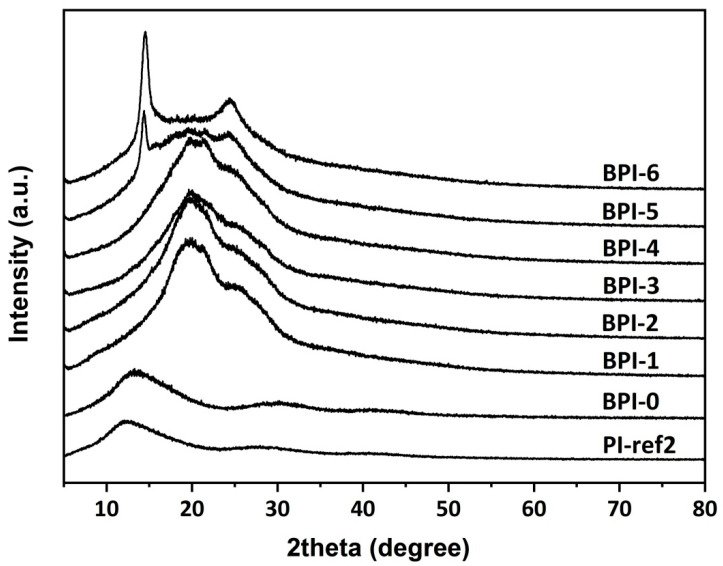
XRD plots of BPI and PI-ref2 films.

At last, it is worth noticing that PI-ref2 (PMDA-NDA) cured at 400 °C also showed quite low WI (1.41) and low Lab values (L* = 2.20; a* = 11.99; b* = 3.33). What is more, according to the simulation results shown in Figure 3, PI-ref2 had a lower E_g_ value than that of BPI-6. Thus, the PI-ref2 film should exhibit higher CT interactions and deeper colors than that of BPI-6. However, in the UV-Vis and CIE Lab measurements, BPI-6 showed the lower optical transmittance and deeper colors. Therefore, the XRD plots of the PI films were further measured, and the results are shown in Figure 8. PI-ref2 showed the typical amorphous structural feature, while BPI-6 showed a clear crystalline nature with the scattering angles in the range of 10~30° due to the ordered packing of the molecular chains in BPI-6 caused by the rigid-rod biphenyl units in the dianhydride moiety and the enhanced molecular chain interactions by the imino (–NH–) units in the diamine moiety. The crystalline domains in BPI-6 efficiently decreased the penetration of visible light and thus endowed the film with deeper colors. Based on the above discussion, one can draw the conclusion that the colors of PI films were simultaneously affected by the CT interactions, crystallinity of the molecular chains, and the processing conditions.

### 3.3. Thermal Properties

**Figure 9:**
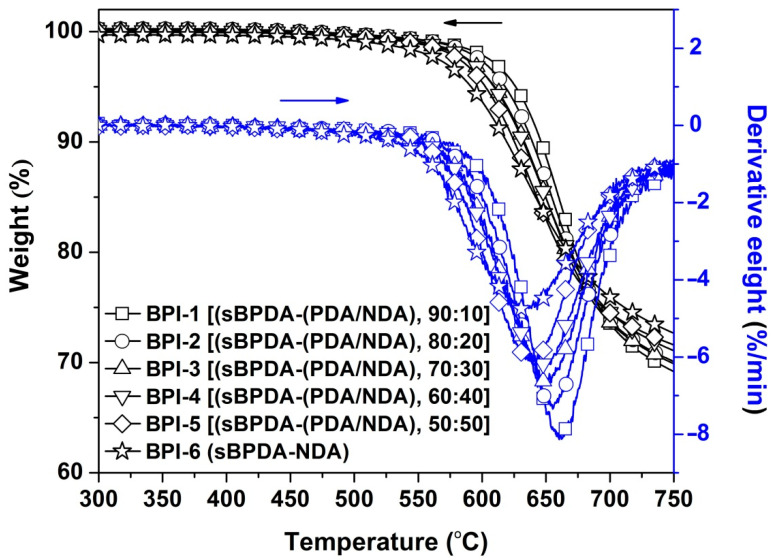
TGA and DTG plots of BPI films in nitrogen.

Various measurements, including TGA, DMA, and TMA, were performed to investigate the structure–thermal properties relationship of the BPI films and the results are summarized in Table 3. First, the TGA and derivative TGA (DTG) plots of the BPI films shown in Figure 9 revealed that the BPI films did not lose the original weights before 500 °C in nitrogen and showed the 5% weight loss temperatures (T_5%_) in the range of 591.3–625.9 °C. All the BPI films showed the residual weight ratios at 750 °C (R_w750_) around 70 wt%. They showed the most rapid decomposition temperatures (T_max_) in the range of 631.3–662.0 °C. All the BPI films showed apparently higher thermal resistance than those of the standard PI-ref1 film. Meanwhile, incorporation of NDA units in BPI-0 matrix decreased the T_5%_ and T_max_ values of the BPI films, increasing the R_w750_ values at the same time. The flexible imino linkages in the NDA units contributed to the inferior initial thermal decomposing temperatures for the polymers. However, the NDA-induced crystallinity for the BPI films increased the weight retentions of the films at elevated temperatures.

**Figure 10:**
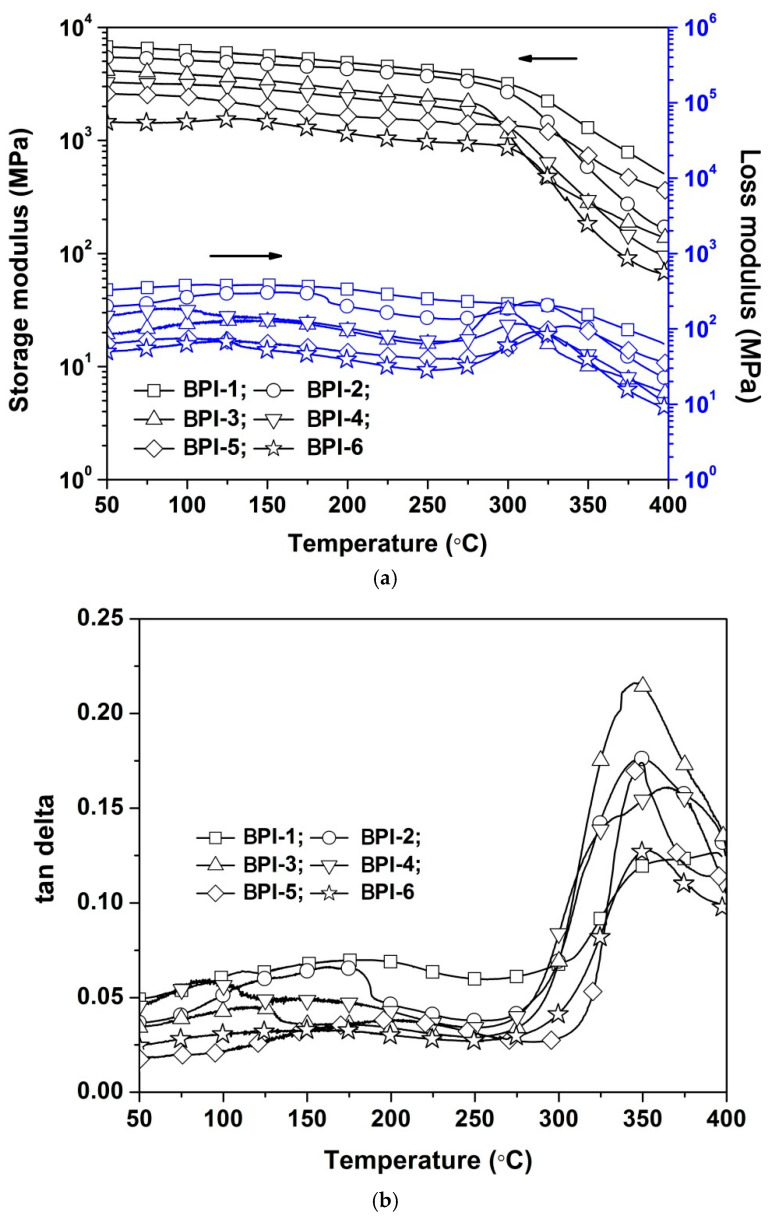
DMA plots of PI films in nitrogen. (**a**) Storage and loss modulus; (**b**) tan delta.

The dynamic mechanical behaviors and glass transition temperatures (T_g_) of the BPI films were evaluated by DMA measurements, and the results are shown in Figure 10. It could be observed from the modulus–temperature plots of the BPI films shown in Figure 10a that incorporation of flexible NDA units gradually decreased the storage modulus (E′) of the BPI films. The BPI-1 showed an initial E′ value of 6.71 GPa at 50 °C, which was obviously higher than that of BPI-6 film (E′ = 1.46 GPa). It was the same trend for the loss modulus (E″) of the BPI films. Apparently, the flexible –NH– linkages in NDA components decreased the modulus of the BPI films. According to the tan delta plots shown in Figure 10b, all the BPI film showed clear glass transition behaviors around 350 °C except for BPI-1. The peaks of the tan delta plots were defined as the T_g_ values of the polymers. Thus, the current BPI films showed the T_g_ values in the range of 347.6–365.5 °C, which were lower than those of PI-ref1 (T_g_ = 418.8 °C) and PI-ref2 (T_g_ = 431.6 °C).

**Figure 11:**
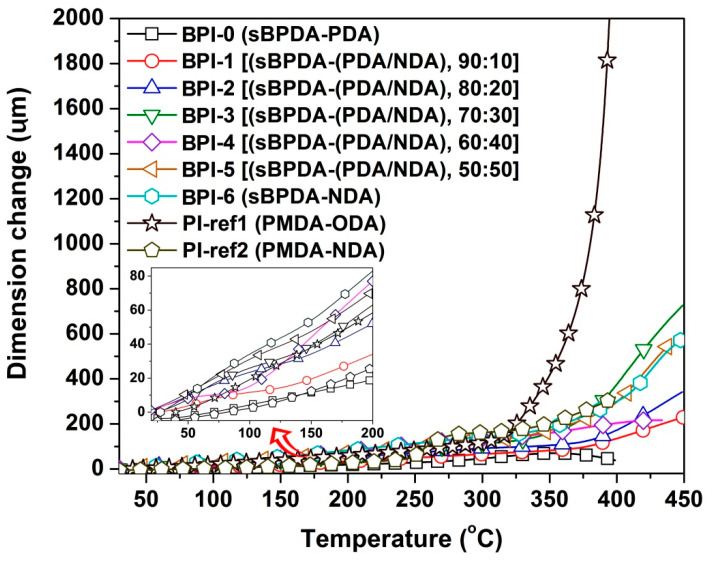
TMA curves of PI and PI-ref films (Insert: Partial TMA plots with the same axis captions with the main plots).

At last, the high-temperature dimensional stability of the BPI films was evaluated by TMA measurements, as shown in Figure 11. BPI-0 film (trademark: Upilex^®^-S) has been well-known for the low coefficient of thermal expansion (CTE) in the wide temperature range and has been widely used in semiconductor industry as substrates for FPCB, COF (chips on films), TAB (tape automated bonding), and other high-tech applications. It could be seen from Figure 11 that BPI-0 film did not show obvious dimensional change in the TMA test and revealed the CTE value of 12.3 × 10^−6^/K in the temperature range of 50–250 °C. With the increasing contents of NDA units in the BPI films, the dimensional stability of the polymers gradually deteriorated. For example, BPI-6 film showed the CTE value of 34.8 × 10^−6^/K, which were apparently higher than those of BPI-0, or even the standard PI-ref1 film (CTE = 29.5 × 10^−6^/K). In the FPCB fabrication, the copper foil has the CTE value around 17.0 × 10^−6^/K. Thus, the PI film substrate should have a similar CTE value in order to prohibit the reliability problems arising from the CTE mismatch in the practical FPCB applications. In the currently developed BPI films, only BPI-1 (CTE = 15.3 × 10^−6^/K) showed a comparable CTE value to that of copper coil. Thus, further decreasing of the CTE values of the BPI films with more deep colors should be further investigated. In our preliminary experiments, incorporation with silica nanoparticles showed good promising results in decreasing the CTE values of BPI-6 film, which will be reported in our next work.

## 4. Conclusions

The current work aimed to develop high-performance BPI films with excellent blackness close to “pure black” level while maintaining good thermal and high-temperature dimensional stability for FPCB applications. Incorporation of the NDA diamine containing chromophoric groups into the intrinsically colored BPI-0 (sBPDA-PDA) system was proven to be effective to achieve the target. The BPI-6 homopolymer derived from sBPDA and NDA showed the dark appearance with the WI of 0.35 and the CIE lab color parameters close to 0. In addition, the BPI-6 film showed good thermal stability, with the initial decomposition temperature higher than 590 °C and T_g_ over 350 °C. Although the BPI-6 film showed a slightly higher CTE value than required, further modification via composite technique might be promising to compensate the gap. The detailed results will be reported in our next work.

## Figures and Tables

**Table 1:** Molecular weights of the PAAs.

Samples	M_n_ ^a^ (×10^4^ g/mol)	M_w_ ^a^ (×10^4^ g/mol)	PDI ^a^
PAA-0	2.73	4.19	1.53
PAA-1	2.75	6.36	2.31
PAA-2	2.80	6.68	2.38
PAA-3	2.90	7.12	2.46
PAA-4	2.93	7.43	2.54
PAA-5	2.93	7.44	2.54
PAA-6	3.04	9.66	3.18

^a^ M_n_: number average molecular weight; M_w_: weight average molecular weight; PDI: polydispersity index, PDI = M_w_/M_n_.

**Table 2:** Optical and thermal properties of the BPI films.

Samples	λ_cut_ ^a^ (nm)	T_550_ ^a^ (%)	T_650_ ^a^ (%)	T_760_ ^a^ (%)	L* ^a^	a* ^a^	b* ^a^	WI ^a^	Haze (%)
BPI-1	415	9.7	40.2	63.4	38.45	35.62	65.74	3.16	1.64
BPI-2	499	5.0	32.7	59.5	27.38	37.70	47.13	5.57	0.40
BPI-3	525	1.3	23.4	53.5	23.78	37.36	40.97	5.75	0.66
BPI-4	529	0.6	14.2	43.3	13.07	31.86	22.51	4.72	0
BPI-5	563	0	5.6	29.5	4.27	22.51	7.34	1.39	0
BPI-6	572	0	1.4	22.5	0.39	2.65	0.66	0.35	1.83
PI-ref1	407	70.4	74.6	80.9	88.65	−9.35	79.41	14.45	0.68
PI-ref2	592 (555) ^b^	0 (0.4)	3.6 (34.8)	36.8 (72.4)	2.20 (23.36)	11.99 (46.43)	3.33 (40.26)	1.41 (1.76)	0 (0)

^a^ λ_cut_: Cutoff wavelength; T_550_, T_650_, T_760_: Transmittance at the wavelength of 550 nm, 650 nm, and 760 nm, respectively; L*, a*, b*: CIE Lab optical parameters, see Measurements; WI: whiteness index. ^b^ The data in the parentheses were for the films cured at 350 °C.

**Table 3:** Thermal properties of the PI and PI-ref films.

PI	T_5%_ ^a^ (°C)	T_10%_ ^a^ (°C)	T_max_ ^a^ (°C)	R_w750_^a^ (%)	T_g_ ^b^ (°C)	CTE ^c^ (×10^−6^/K)
BPI-0	ND ^d^	ND	ND	ND	ND	12.3
BPI-1	625.9	646.1	662.0	69.2	ND	15.3
BPI-2	617.8	639.3	655.3	70.1	349.5	20.9
BPI-3	609.6	632.8	649.5	69.9	347.6	26.6
BPI-4	609.3	632.2	646.8	71.1	365.5	28.9
BPI-5	602.4	625.3	642.3	71.6	349.0	32.2
BPI-6	591.3	619.1	631.3	72.7	351.7	34.8
PI-ref1	581.0	594.8	605.1	61.6	418.8	29.5
PI-ref2	515.7	559.6	591.3	59.6	431.6	18.8

^a^ T_5%_, T_10%_: Temperatures at 5% and 10% weight loss, respectively; T_max_: Temperature at which the rapid decomposition was recorded; R_w750_: Residual weight ratio at 750 °C in nitrogen; ^b^ T_g_: Glass transition temperature detected by DMA measurements; ^c^ CTE: linear coefficient of thermal expansion in the range of 50–250 °C; ^d^ Not detected.

## Data Availability

Data are contained within the article.
